# Statistical analysis of the Bacterial Carbohydrate Structure Data Base (BCSDB): Characteristics and diversity of bacterial carbohydrates in comparison with mammalian glycans

**DOI:** 10.1186/1472-6807-8-35

**Published:** 2008-08-11

**Authors:** Stephan Herget, Philip V Toukach, René Ranzinger, William E Hull, Yuriy A Knirel, Claus-Wilhelm von der Lieth

**Affiliations:** 1 Core Facility: Molecular Structure Analysis (W160), German Cancer Research Center, Heidelberg, Germany; 2 N.D. Zelinsky Institute of Organic Chemistry, Russian Academy of Sciences, Moscow, Russia

## Abstract

**Background:**

There are considerable differences between bacterial and mammalian glycans. In contrast to most eukaryotic carbohydrates, bacterial glycans are often composed of repeating units with diverse functions ranging from structural reinforcement to adhesion, colonization and camouflage. Since bacterial glycans are typically displayed at the cell surface, they can interact with the environment and, therefore, have significant biomedical importance.

**Results:**

The sequence characteristics of glycans (monosaccharide composition, modifications, and linkage patterns) for the higher bacterial taxonomic classes have been examined and compared with the data for mammals, with both similarities and unique features becoming evident. Compared to mammalian glycans, the bacterial glycans deposited in the current databases have a more than ten-fold greater diversity at the monosaccharide level, and the disaccharide pattern space is approximately nine times larger. Specific bacterial subclasses exhibit characteristic glycans which can be distinguished on the basis of distinctive structural features or sequence properties.

**Conclusion:**

For the first time a systematic database analysis of the bacterial glycome has been performed. This study summarizes the current knowledge of bacterial glycan architecture and diversity and reveals putative targets for the rational design and development of therapeutic intervention strategies by comparing bacterial and mammalian glycans.

## Background

Natural glycans are known to take part in many key biological processes such as cell adhesion, recognition, receptor activation or signal transduction, and they also exhibit major structural functions in both bacteria and plants. In addition, bacterial glycans act as virulence, osmoprotection and desiccation protection factors [1]. The diversity of structures within the mammalian glycome seems limited and is well described in the literature [2]. On the other hand, the bacterial glycome exhibits greater diversity, stemming largely from the distinct cell wall architecture of these organisms.

The cell envelope of either Gram-positive or Gram-negative bacteria is based on peptidoglycan, a polymer in which polysaccharide chains are cross-linked with short peptide chains [3]. Gram-negative bacteria possess an additional outer membrane that is composed of a lipopolysaccharide-protein complex. Gram-positive bacteria have no outer membrane, but the peptidoglycan wall is thicker (> 30 nm vs. 10 nm in Gram-negative bacteria) and contains polysaccharides with teichoic acids attached (a carbohydrate polymer containing alditols and phosphodiester linkages).

Both Gram-positive and Gram-negative bacteria produce extracellular polysaccharides, present either as a discrete capsule covalently attached to the cell envelope or as a slime weakly bound to the cell surface. These various glycoconjugates and polysaccharides on the surface of the cell often contain the antigenic determinants that initiate an immunogenic response in a host. In addition, these surface carbohydrates provide recognition elements for pathogens such as bacteriophages.

The lipopolysaccharide of Gram-negative bacteria contains lipid A, a phosphorylated GlcN-GlcN disaccharide moiety, *N*- and *O*-acylated with fatty acids which anchor the molecule in the outer leaflet of the outer membrane. Lipid A is covalently linked to a heteropolysaccharide which interacts with the environment and consists of an inner core (commonly containing Kdo (3-deoxy-D-*manno*-oct-2-ulosonic acid) and *manno*-heptoses) and an outer *O*-specific chain, a complex polysaccharide which determines the serological or antigenic properties of the lipopolysaccharide [4,5]. These so-called *O*-antigens are mainly heteropolymers containing a large variety of residues (mainly monosaccharides, but also alditols, amino acids, etc.). These components, together with the capsular polysaccharides (K-antigens [6,7]), can elicit an immune response in higher organisms.

The structures of the various carbohydrate antigens are unique, often being characterized by repeating units in the polymer structure. Indeed, all types of monosaccharides, including L-rhamnose (6-deoxy-L-mannose) and L-fucose (6-deoxy-L-galactose), are found in bacteria, together with rarer, modified sugars, such as 3,6-dideoxyhexoses and Kdo. Knowledge of the structures of surface carbohydrates and their variations is required for understanding how cellular recognition, adhesion, and the immune response operate at the molecular level. This understanding provides a basis for the design of synthetic carbohydrate-based vaccines, diagnostic agents, and immunostimulators. Certain fragments of bacterial polysaccharides, in the form of appropriate glycoconjugates, are known to act as vaccines [8].

Carbohydrates represent the most diverse class of biopolymers, and there is growing interest in the study and analysis of this diversity and its biomedical significance. For example, vertebrate glycan variability is assumed to act as a barrier that prevents the spread of an infection within a given population [9]. Although it is widely known that the diversity of carbohydrates is much greater in bacteria than in mammals, no systematic attempt has been undertaken to examine the diversity of bacterial carbohydrates in detail. The structures deposited in glycoscience databases have been only sporadically evaluated. However, statistical structure-oriented investigations using carbohydrate databases were proven to be useful for immunochemical research and serotyping [10]. Systematic analysis of all publicly available data will not only expand our general knowledge and understanding of the complexity of glycans in biological systems but will also offer a framework for the design of more comprehensive high-throughput screening methods or devices.

Comprehensive data concerning carbohydrate diversity within the entire bacterial world will be useful for the classification of bacteria according to their glycan structures and facilitate the search for the most widespread carbohydrate markers of various bacterial taxonomic groups. These markers are critical for medical applications, and a simple ranking by abundance is a good starting point for the design of synthetic biologically-active carbohydrates and for corresponding immunological studies. In particular, the statistics of *monomer composition *reveal potential taxonomic markers and also simplify the creation of carbohydrate microarrays by providing candidates for spotting [11].

A one-enzyme-class/one-saccharide-linkage paradigm applies for almost all individual steps of glycan biosynthesis. Accordingly, complete information on the *diversity of disaccharide fragments *allows one to describe the diversity of the glycosyltransferases expressed in individual taxonomic groups, and these enzymes may become potential targets for antimicrobial treatment.

For this study we performed statistical analyses of the Bacterial Carbohydrate Structure Data Bank (BCSDB), the largest database for bacterial glycans containing nearly all known bacterial glycan structures published up to 2007 [12]. For comparison the mammalian glycans documented in the GLYCOSCIENCES.de database [13] (derived mainly from CarbBank [14]) have also been examined. The properties analyzed include glycan size, branching, and charge density, as well as the frequency of occurrence of specific monosaccharide residues, residue pairs and their linkage configurations. Precise definitions for the terminology used in this study can be found in the Methods section.

## Results and Discussion

### Distribution of carbohydrate structures among taxonomic groups

We first examined the number of sequences found in the BCSDB and GLYCOSCIENCES.de for various taxonomic ranks (class, order, family). Where possible, the taxonomic relationships were traced using the NCBI taxonomy database [15]. The GLYCOSCIENCES.de database currently contains a total of 23120 glycan and glycoconjugate records, of which 13704 records for diverse animal, plant, bacteria and fungi classes have some information concerning taxonomy. In the BCSDB there are a total of 8504 records for bacteria only, and 8479 of these contain information concerning taxonomy. These numbers may include multiple records for a given glycan when the same glycan is reported for more than one species. Note that not all taxonomic classes are represented in the databases and that for bacterial glycans there is considerable overlap between the two databases.

**Figure 1:**
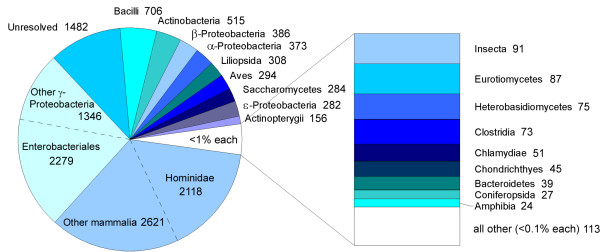
**Distribution of carbohydrate sequences for various taxonomic classes**. For the combined BCSDB and GLYCOSCIENCES.de databases the pie chart sector areas correspond to the distribution (in percent) of the 13775 assigned sequences within the taxonomic classes shown, while the labels give the absolute numbers of sequences. The white pie sector contains all classes which each have < 1% of the total assigned records. This category is expanded in the bar chart at the right, where the bottom block "all other" contains all classes which each have < 0.1% of the assigned records. Class names ending with ...*opsida *or ...*mycetes *correspond to plants or fungi, respectively; *Actinopterygii *contains fish while *Chondrichthyes *contains sharks.

If we now consider the two databases combined, there are a total of 13775 nonredundant carbohydrate records which include taxonomic information. The distribution of these records among various taxonomic classes is shown both numerically and schematically in Fig. 1. A more detailed breakdown of the distribution can be found in the additional material [see Additional file 1].

The taxonomic class *Mammalia *is found to have 4739 assigned sequence/taxon pairs, of which 2118 are of human origin (family *Hominidae*). All other animal or plant classes in the database have less than 350 pairs. The category "unresolved" refers to the 1482 records for which the source is defined but the specific taxonomic class could not be traced automatically using the NCBI. Only about half of the bacterial phyla are represented in the BCSDB with a total of 6098 sequence/taxon pairs, and nine classes have less than 10 records. Note that the number of carbohydrates or glycoconjugates registered for a given taxonomic class does not necessarily reflect its species diversity, but more likely the intensity with which the class has been studied. Thus, the apparent diversity of carbohydrates in the various taxonomic classes reflects to a large part the information bias in the published literature, and this situation must be kept in mind when making conclusions based on the distributions of properties discussed below.

In the combined databases there are a total of 12659 records in the category "no taxonomy" which means that either no information concerning the taxonomy of the source is available or that the carbohydrate is not of purely natural origin. These records were not included in Fig. 1 and were not used in the following analyses.

### Choice of taxonomic datasets for statistical comparisons

For the following more detailed statistical comparisons, we defined two sets of taxonomic *groups*, considering both biological and coverage aspects. Taxonomy Set 1 (Table 1) was defined to provide an overview of the total content of the two databases used for the general comparison of bacterial and mammalian carbohydrates, taking into account the fact that bacterial carbohydrates frequently contain repeating units while mammalian sequences usually do not. Thus, Set 1 contains three taxonomic groups: all mammalian carbohydrates, all bacterial carbohydrates with nonrepeating sequences (oligomers), and all bacterial sequences with repeating units (polymers).

**Table 1:** Definition of taxonomy Set 1.

**Group Name**	Number of sequences*	**Explanation**
Mammalia (class)	3328	Total number of different carbohydrate (glycan) sequences for Mammalia, as registered in GLYCOSCIENCES.de.
Bacteria (polymers)	2250	Total number of different repeating units in the polymeric carbohydrate (glycan) sequences for all bacteria registered in the BCSDB.
Bacteria (oligomers)	3210	Total number of different oligosaccharide sequences (nonrepeating units) for all bacteria registered in the BCSDB.

* For each group each unique carbohydrate sequence was counted only once; a given sequence may occur in more than one taxonomic group.

For comparisons within the taxonomic domain *Bacteria*, we defined a more detailed taxonomy Set 2 (Table 2), which includes two classes of Gram-positive bacteria (*Actinobacteria *and *Bacilli*) and the various classes of the phylum *Proteobacteria*. The largest of these classes, the γ-*Proteobacteria*, has been further subdivided in Set 2 into the major order *Enterobacteriales *and a subset containing all other γ-*Proteobacteria*. The class δ-*Proteobacteria *(with only 2 records) has been combined with the ε-*Proteobacteria*.

**Table 2:** Definition of taxonomy Set 2.

Bacterial Class*	Number of sequences**	**Gram reaction**
*Actinobacteria*	395	+
*Bacilli*	640	+
α-*Proteobacteria*	324	-
β-*Proteobacteria*	365	-
(γ-*Proteobacteria*)	(3305)	-
*Enterobacteriales *[order]	2087	
other γ-*Proteobacteria *[orders]	1218	
δ/ε-*Proteobacteria*	284	-
(δ-*Proteobacteria*)	(2)	
(ε-*Proteobacteria*)	(282)	

* The groups used to define Set 2 are those with more than 200 sequences and are listed without parentheses.

** For each class each unique carbohydrate sequence was counted only once; a given sequence may occur in more than one taxonomic group.

In order to obtain meaningful statistics, only those taxonomic groups are compared for which at least 200 carbohydrate sequences are available. For this reason the classes Chlamydiae, Clostridia, and Bacteroidetes, for example, have not been included in Set 2. Note that in Tables 1 and 2 the total number of *unique *carbohydrate *sequences *in each group is listed, and these sets were utilized in all subsequent analyses.

### Carbohydrate size, branching and charge density

**Figure 2:**
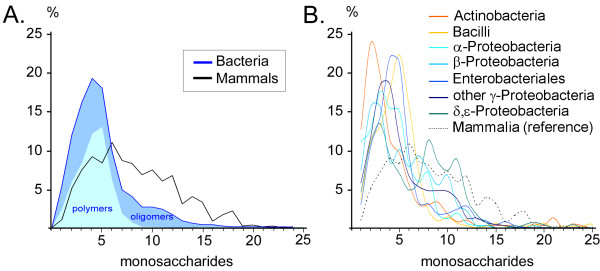
**Size distribution of carbohydrate sequence units**. The normalized frequency distribution for total carbohydrate residue count per sequence unit is shown in percent of total sequences for each taxonomic group. **A**. For taxonomy Set 1 the solid blue curve represents the cumulative values for bacterial oligomers (blue-shaded region) and the repeating units of polymers (cyan region) in comparison with mammals (black curve). **B**. The frequency distribution for carbohydrate residue count per sequence unit is shown for each of the bacteria groups defined in taxonomy Set 2, using the color coding defined in the legend. For comparison the dotted curve shows the distribution for mammals. The curves are smoothed for visual clarity.

Frequency distributions for general measures of molecular size, topology (branching) and mean charge density have been calculated for the carbohydrate sequences comprising the various taxonomic groupings described by Set 1 and Set 2 (Tables 1 and 2). In each case the distributions are normalized to the *total number of sequences in each taxonomic group *and expressed as percentages within each group. In Fig. 2A distributions for the number of monosaccharides per sequence *unit *(either the entire carbohydrate sequence for oligomers or the repeating unit for polymers, see Definition 4 in the methods) are shown for bacteria vs. mammals (taxonomy Set 1). The distribution is relatively broad for mammals with mean and median values, respectively, of 8.17 and 8 monomers per sequence, while for bacteria the distribution shows a narrow peak at 4–5 monomers for both oligomers (mean: 5.94 median: 5) and for the repeating unit of polymers (mean: 4.17, median: 4). However, oligomers show a significant population of sequences with 8–15 monomers while the distribution for polymers essentially ends at 9 monomers per unit. Of course, the total length of a polymeric sequence with multiple units may very well exceed the maximum length of oligomers. Naturally occurring oligomers may also be longer than the sequences reported in the databases since the process of extracting and isolating a glycan may result in partial digestion and loss of residues.

In Fig. 2B the distributions of the size parameter for the bacterial groups defined in taxonomy Set 2 are found to differ considerably. Narrow distributions with essentially a single prominant peak are found for *Actinobacteria *(mean: 4.51, median: 3), *Bacilli *(mean: 5.18, median: 5) and the order *Enterobacteria *(mean: 5.18, median: 6) with peaks at ca. 2.5, 5.5 and 4.5 residues, respectively. The various other classes of *Proteobacteria *have broader distributions with more or less pronounced multiple peaks, e.g. at 3, 8 and 11 residues for the δ,ε-Proteobacteria group.

**Figure 3:**
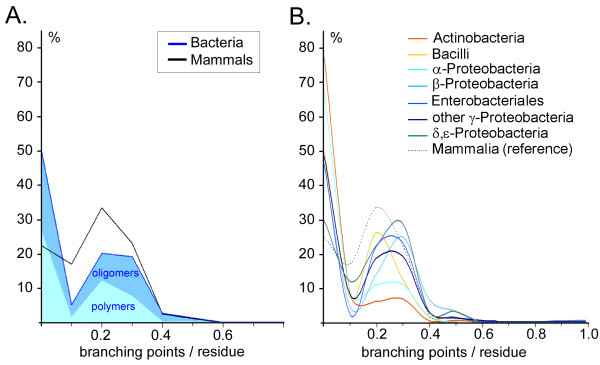
**Branching index distribution**. The normalized frequency distributions for the number of branching points *per residue *are shown for the carbohydrate sequence units of taxonomy Set 1 (**A**) and Set 2 (**B**), analogous to the graphs in Fig. 2.

The number of branching points per carbohydrate residue can be considered to be a *branching index *which reflects the complexity of carbohydrate topology. Fig. 3A demonstrates that 22% of all mammalian and 50% of all bacterial sequences are linear (branching index = 0). However, for the individual bacterial groups of taxonomy Set 2, the percentage of linear structures ranges from 30% to 78% (Fig. 3B). A general feature of all branching point graphs in Fig. 3 is a peak in the distribution at a branching index of 0.2 – 0.3, which corresponds to carbohydrate sequences with one branching point for every three to five monosaccharide residues. This peak in the distribution is weak for *Actinobacteria *and α-*Proteobacteria *but strong for mammals, *Bacilli*, and other *Proteobacteria*.

**Figure 4:**
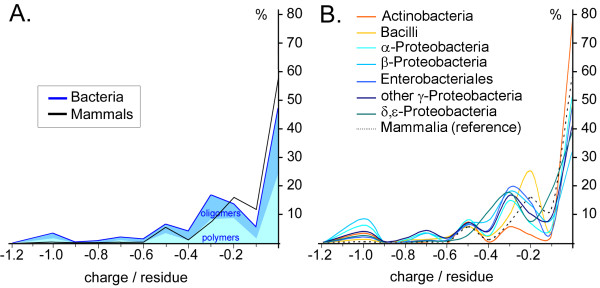
**Mean charge density distribution**. Normalized frequency distributions for the maximum possible mean charge density per residue are plotted for the carbohydrate sequence units of taxonomy Set 1 (**A**) and Set 2 (**B**), analogous to the graphs in Fig. 3.

Finally, the *mean charge density *parameter (max. electric charge possible for all ionizable groups divided by the number of carbohydrate residues in a sequence unit) is shown in Fig. 4 for taxonomy Set 1 and Set 2. About 58% of mammalian sequences and 47% of all bacterial carbohydrate sequence units have no net charge (Fig. 4A). For the bacterial groups of Set 2, the frequency of sequence units with no net charge ranges from ca. 32% for β-*Proteobacteria *to 77% for *Actinobacteria *(Fig. 4B). All other sequences have a net negative charge due to carboxyl, phosphate or sulfate groups, for example. The distributions for mammals and *Bacilli *both have peaks at charge densities of -0.2 and -0.5; the δ,ε-*Proteobacteria *distribution exhibits a single broad peak at ca. -0.3 while the other bacterial distributions have multiple peaks at -0.3, -0.5, -0.7 and -1.0 (Fig. 4B).

### Monosaccharide diversity

For mammals and even more so for bacteria, the diversity of the monosaccharides used as the building blocks of carbohydrate sequences is significantly larger than that for the residues in proteins or nucleic acids. From the GLYCOSCIENCES.de database a total of 35 different monosaccharides were found for mammalian carbohydrates, according to the nomenclature used in the original databases (Table 3). This degree of diversity is at first glance puzzling, in view of the common notion that mammalian carbohydrates are built up of 10 "classical" monosaccharides (Glc, Gal, GlcNAc, GalNAc, Man, GlcA, Fuc, Neu, IdoA, Xyl) [1]. However, the variety of monosaccharides defined in the primary databases is higher due to (a) residues being specified with unknown anomer or ring type definitions, (b) analytical artifacts from the structure elucidation process (alditols, double bonds), or (c) secondary modifications such as sulfation. Furthermore, carbohydrate sequence databases are not error-free and suffer from incorrect structure elucidations and curation mistakes. Since the existing databases generally use free-text identifiers for the monosaccharides, it was helpful to translate all structural database entries into a machine-readable notation called GlycoCT [16]. Using structural filters based on this notation we were able to significantly reduce the fuzziness introduced by the lack of strictness in the definitions of the original sequences (Table 3). During the analysis we excluded manually common artifacts caused by analytical procedures and entries with undefined absolute or anomeric configuration or ring type.

**Table 3:** Diversity of monosaccharides, basetypes and basic entities for various taxonomic groups.

**Group**	**Monosaccharides**	**Basetypes**	**Basic entities**
*Mammalia*	35	14	10 *
*Bacteria*	551	143	123
*Actinobacteria*	100	48	33
*Bacilli*	100	41	34
α-*Proteobacteria*	68	34	26
β-*Proteobacteria*	69	33	32
*Enterobacteriales *(γ-*Proteobacteria*)	243	76	65
other γ-*Proteobacteria*	203	70	76 **
δ/ε-*Proteobacteria*	52	35	32

The total number of different monosaccharides, basetypes, and basic entities are listed for each group in taxonomy Set 1 and Set 2. When counting in the combined databases, an occurrence threshold of 10 for mammals and 2 for bacteria was employed.

* The basic entity D-Fuc was excluded from *Mammalia *for reasons given in the text.

** In this case there are more basic entities compared to basetypes because amino derivatives are included in the basic entities.

To minimize the influence of errors and artifacts on the statistics of Table 3, a threshold for the occurrence of monosaccharides, basetypes and basic entities was set at 10 for mammals and 2 for bacteria. This means that a given residue type was included in the statistics only when its number of occurrences exceeded the defined threshold. A relatively low threshold was chosen for bacteria because, in contrast to mammals, bacteria are known to produce a great variety of unique monosaccharide residues with low occurrence. When the threshold for bacteria was reduced from 2 to 0, the diversity of detected residues increased by about 25%. A complete list of monosaccharide residues and basetypes found for each taxonomic group is available in GlycoCT nomenclature in the additional material [see Additional files 2 (tables a-i) and 3].

For mammals the analysis returned 18 occurrences of D-Fuc as a basic entity. However, this residue was excluded from Table 3 because all carbohydrates in GLYCOSCIENCES.de which are specified to contain D-Fucose originate from old publications in which the absolute configuration of Fuc was not specified. The occurrence of D-Fuc in mammalian carbohydrate records can be regarded as a data translation error since there is no evidence for a mammalian enzyme which synthesizes D-Fuc.

**Figure 5:**
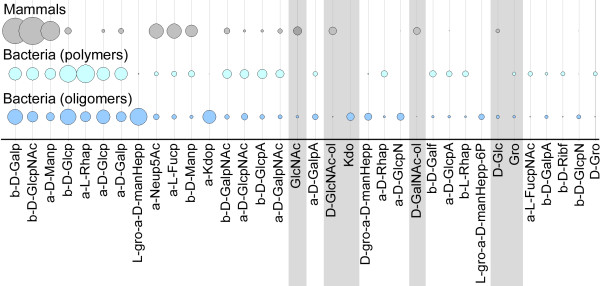
**Diversity of monosaccharides in bacteria and mammals**. For taxonomy Set 1 the 35 most abundant monosaccharide residues are listed from left to right in decreasing order of their total abundance across all taxonomic groups. The three rows of circles correspond to the three groups: mammals (gray), bacteria (polymers, cyan), bacteria (oligomers, blue), as defined in Table 1. Circle areas reflect the relative abundance of a monosaccharide residue within each group (monosaccharide count/total residue count per taxonomic group). Residues that are the result of analytical artifacts or incomplete structure elucidation (hexosamine alditols, glycerol, D-Glc, etc.) are highlighted with gray bars. In this and subsequent Figures the anomeric designators α and β are written as a and b, the ring type designators *p *and *f *are shown as p and f.

The 35 monosaccharides with the highest occurrence within taxonomy Set 1 are shown schematically in Fig. 5. Of them, all 17 monosaccharides that were found in mammalian carbohydrates are also found in the bacterial world. Rhamnose, L-*glycero*-α-D-*manno*-Heptose, α-D-Galacturonic acid and α-Kdo are the most frequent monosaccharides that are unique to bacteria and, except for Rhamnose, are preferably located in the core portions of bacterial saccharides, in accordance with the typical lipopolysaccharide (LPS) structure of Gram-negative bacteria, the classes which dominate in this analysis (Table 2).

**Figure 6:**
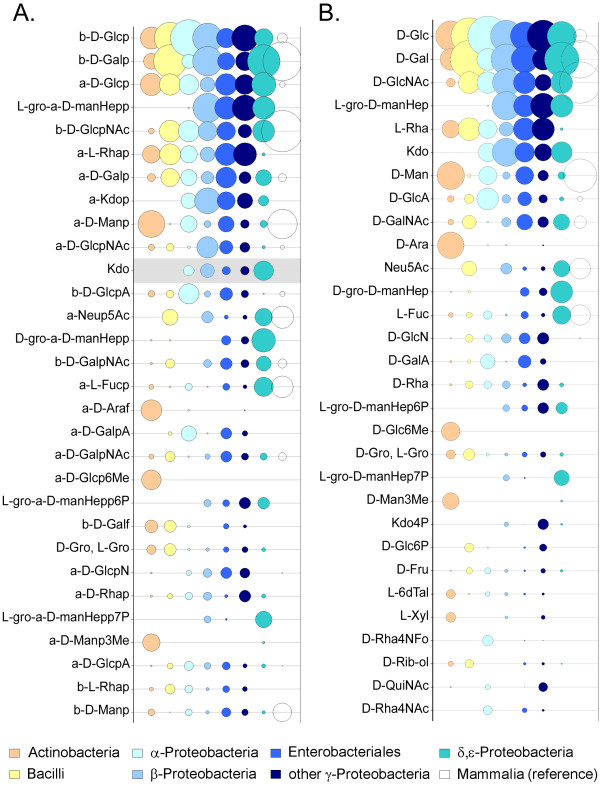
**The most abundant monosaccharides in bacteria**. Circle areas reflect relative abundances of the 30 most common monosaccharide residues (**A**) or basic entities (**B**) for the carbohydrates found in the BCSDB for each of the bacterial taxonomy groups of Set 2. Within each group the abundances are normalized to the total number of residues per group. The color code (see legend at bottom) is the same as in Figs. 2–4; for comparison, the open circles represent data for mammals. The residues are sorted from top to bottom in order of decreasing total abundance *in bacteria *(order differs from Fig. 5). The residue Kdo (without anomeric configuration) results from analytical artifacts and is highlighted in gray. For the basic entities defined in **B**, no distinction is made between anomeric configurations and ring types.

A more detailed analysis of monosaccharide residues in the bacterial taxonomy groups of Set 2 is shown in Fig. 6. Kdo and L-*glycero*-D-*manno*-Heptose are confined to Gram-negative bacteria, whereas Gram-positive bacteria seem to have an excess of arabinoses and methylated hexoses.

**Figure 7:**
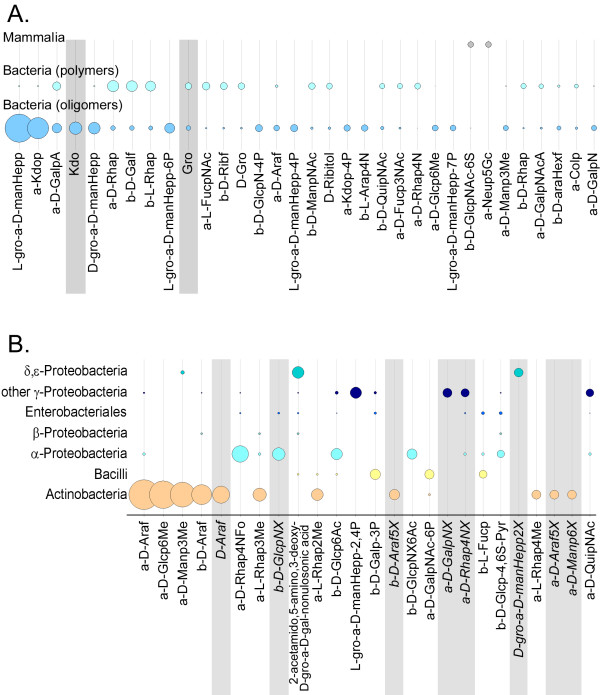
**The most abundant unique monosaccharides**. Circle areas reflect relative abundances within a taxonomic group for those unique monosaccharide residues which appear exclusively, or nearly so, in a single taxonomic group of Set 1 (**A**) or Set 2 (**B**). Uniqueness is defined here as: frequency in the selected group > 0.1%, frequency in other groups < 0.1%. Residues that result from analytical artifacts and those that are ambiguous due to incomplete structure elucidation are highlighted in gray. The symbol *X *represents any substituent.

Generally, monosaccharides that are unique to bacteria are of special interest as potential immunogenic targets. Existing vaccines frequently take advantage of the unique saccharides in the complex carbohydrates located on the surface of bacteria [11]. Fig. 7 presents the unique monosaccharides (see definition of *unique *in the Figure legend) found for the bacterial and mammalian groups. Due to the greater diversity of bacterial monosaccharides, many carbohydrates unique to the bacterial world were found (especially for Gram-positive bacteria), whereas only two mammalian monosaccharides [α-*N*-Glycoloylneuraminic acid (α-Neu5Gc) and β-D-*N*-acetylglucosamine-6-*O*-sulfate (β-D-GlcpNAc-6S)] appear to have no counterpart in the bacterial world. It is known that neuraminic acid derivatives are typically found at the terminal positions of mammalian glycoconjugates, being mediators for cell-cell interaction or receptors for pathogens [17]. The presence of exposed α-Neu5Ac residues in bacteria may be an evolutionary advantage through which bacteria mask themselves to the host immune system.

Attention should be paid to the distribution of monosaccharides at the terminal positions of oligomers and side chains of polymers. In higher organisms such residues are optimally positioned to mediate recognition by endogenous carbohydrate-binding proteins [9]. According to our findings bacterial carbohydrates often have glucose residues at the nonreducing ends, in contrast to mammalian glycans (data not shown). This may be the result of the evolutionary adaptation of bacteria since exposed terminal glucose residues are important for the adherence of bacteria and entry into host epithelial cells, as demonstrated for *Salmonella *and *Pseudomonas *[18].

**Figure 8:**
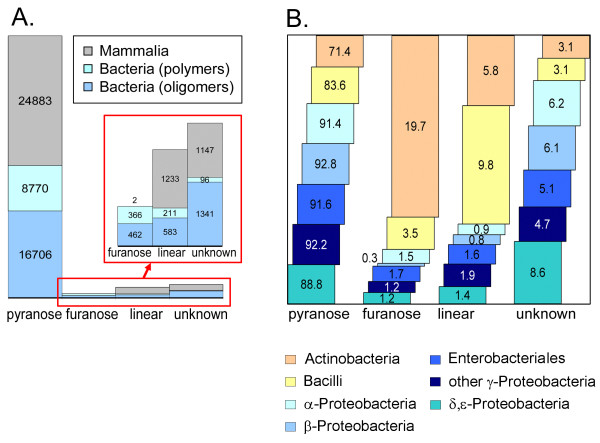
**Monosaccharide ring type distributions**. The distribution of residue ring type (pyranose, furanose, linear, unknown) is shown schematically for various taxonomic groups. **A**. For taxonomy Set 1 the areas of the colored bars are proportional to the absolute occurrences (numbers shown) of a given ring type in each taxonomic group. The vertical scale is expanded in the inset. **B**. For taxonomy Set 2 two different ways of viewing the data are presented. (1) For a given ring type the *area *or *height *of each colored bar in a stack represents the relative abundance (%) of that ring type for each of the taxonomic groups, normalized to the total occurrence of that ring type across all groups (stack height = 100% for each ring type). Thus, the bar heights within a stack represent the distribution of a single ring type across all taxonomic groups. (2) The number labeled in each bar represents the frequency (in %) of residues with the corresponding ring type within the bar's taxonomic group, normalized to the total number of residues for that group. The numbers sum horizontally to 100% for each taxonomic group (color) and, therefore, represent the distribution of the different ring types within an individual group.

Fig. 8 demonstrates that more than 70% of the monosaccharides in every taxonomic group are reported to be in the pyranose form, with most groups even reaching 90%. An interesting finding is that more than 50% of all furanose residues found in bacteria are in the 395 glycan sequences of the class *Actinobacteria *(cf. area of bars in Fig. 8B). Nearly 20% of all residues in *Actinobacteria *glycans are in the furanose form, compared to < 4% for all other bacterial groups studied. A high proportion of furanose residues has also been found for plants (data not shown). In the majority of cases where linear forms or rings of unknown size are found, they can be explained as artifacts of the structure elucidation process, especially when present at the reducing end. However, linear monosaccharides are known to occur occasionally in bacterial carbohydrate sequences and are most prevalent in *Bacilli *and *Actinobacteria *(Fig. 8B).

### Monosaccharide modifications

**Figure 9:**
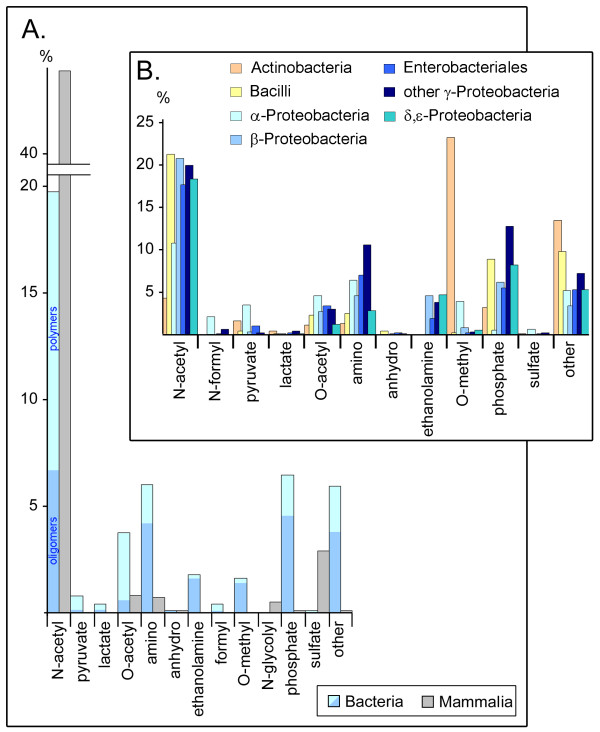
**Distribution of monosaccharide modifications**. Frequency distributions (in %) for secondary modifications of monosaccharide residues are shown for taxonomy Set 1 (**A**) and Set 2 (**B**), normalized to the total number of carbohydrate residues within each taxonomic group. In **A **the bars for bacterial oligomers (blue) and polymers (cyan) are stacked to give the cumulative values for all bacteria studied.

Part of the diversity of monosaccharides can be found in their modifications (Fig. 9). For bacteria secondary modifications often play a role in the mediation of reactivity and lability to various environmental conditions such as pH. The *N*-acetylamino group is the most common substituent for carbohydrates in mammals (ca. 45% of all residues) and in most bacteria classes (ca. 18–21% of all residues), except for *α-Proteobacteria *(11%) and *Actinobacteria *(4.5%). Acetylation of amino groups plays a key role in regulating the ability of amino sugars to form hydrogen bonds and to bear charge [19].

*O*-methylation is the most frequent modification for *Actinobacteria *(ca. 23% of all residues, mainly at O6 of glucose) but occurs with a frequency of < 5% in other bacteria classes and is essentially absent in mammals.

*O*-acetyl, amino, or phosphate substituents are also much more prevalent for bacteria (4–7%) than for mammals (< 1%), and the *O*-acetylation pattern is often different for different cultures of a single bacteria strain. *O*-acetyl groups mask the protective epitopes for bacteria through steric hindrance or altered conformations, as shown for *Meningococci *[20].

Amino sugars with free aminogroups are present in about 7% of bacterial carbohydrate residues compared to ca. 1% for mammals and feature a positively charged -NH_3_^+ ^substituent at neutral pH. The occurrence of these residues in the bacterial cell wall affects hydrophobicity and makes bacteria resistant to the lysozyme of the host, as has been demonstrated for glucosamine in Gram-positive bacteria [21]. Several secondary modifications appear to be unique for bacterial carbohydrates (pyruvate, lactate, ethanolamine, *O*-methyl and formyl) while sulfation or *N*-glycolyl substitution occurs primarily in mammals. Finally, about 7% of bacterial residues have modifications listed under the category "other" in Fig. 9A, with *Actinobacteria *and *Bacilli *showing the highest frequencies (Fig. 9B).

### Disaccharide fragment patterns in bacteria and mammals

**Figure 10:**
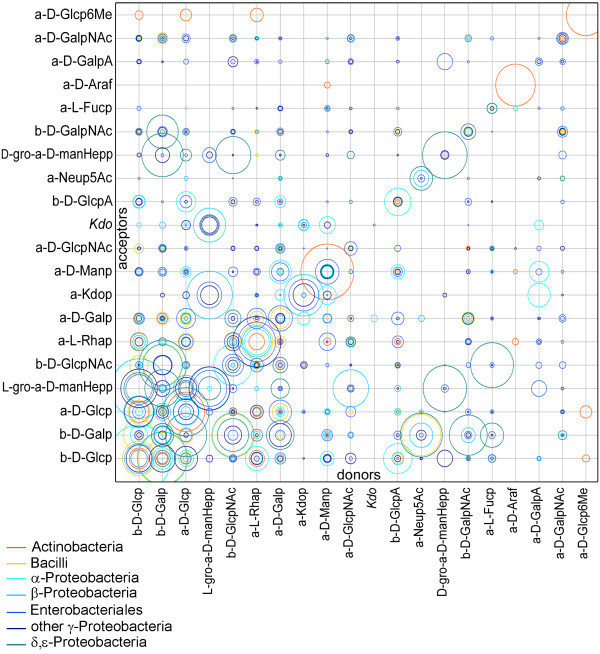
**Distribution of monosaccharide pairs in various bacterial groups**. For taxonomy Set 2 the matrix presents relative abundance data for monosaccharide residue pairs of all linkage types, involving the 20 most common residues serving as donor (children) or acceptor (parent). Each circle area reflects the relative abundance of a given donor-acceptor pair (matrix coordinates) within the corresponding taxonomic group, normalized to the total number of pairs within that group.

The topological characteristics of glycan architecture can be described by statistics which document the frequency distributions for specific neighboring pairs of monosaccharides connected either with *any *type of linkage (monosaccharide pair analysis) or via *specific *linkage positions (disaccharide pattern analysis). The matrix diagram in Fig. 10 illustrates the statistics of linked monosaccharide pairs for bacteria. Here the frequencies of any type of glycosidic linkage between the 20 most common donor and acceptor residues are shown. The areas of the circles plotted at the coordinates for a given pair represent its relative abundance within a given bacterial taxonomic group. Note that not all possible monosaccharide pairs are actually found in the natural sequences registered in the database (missing circles). Some combinations are exclusive for Gram-positive bacteria, e.g. those involving α-D-Ara*f *or α-D-Glc*p*6Me in *Actinobacteria *while others may exhibit similar or widely differing abundances across the taxonomic groups. The high abundances found along the diagonal of the matrix stem from homopolymeric subsequences which are frequent in bacteria. Note that the results for "Kdo" (without anomeric configuration) originate from analytical artifacts. Detailed results for a total of 676 pairs in bacterial and mammalian carbohydrates are summarized in the additional material section [see Additional file 4].

In order to describe carbohydrate sequences at a higher level of complexity, we need to consider not only the identities of the linked monosaccharides but also the linkage configuration. All free hydroxyl groups on each acceptor monosaccharide are potential sites of glycosyltransferase reactions. Therefore, we define the child (donor) to parent (acceptor) connection in terms of the directed glycosylation linkage pattern, analogous to reaction patterns described elsewhere [22]. Thus, the descriptor "a1–4", for example, indicates that an alpha anomeric O1 of the donor is linked to C4 of the acceptor. The statistics of linkage patterns provide a direct description of the expression and activity of glycosyltransferases and the carbohydrate structure repetoire in an organism or taxonomic group. Such statistics have been employed successfully for a variety of bioinformatic tasks with the glycome, e.g. matrix generation [23] and pattern detection [24]. This kind of information is also valuable for recognizing both unique and common linkages and can serve as a basis for a deeper understanding of the immunogenicity of bacterial carbohydrates and for designing targeted vaccines.

**Figure 11:**
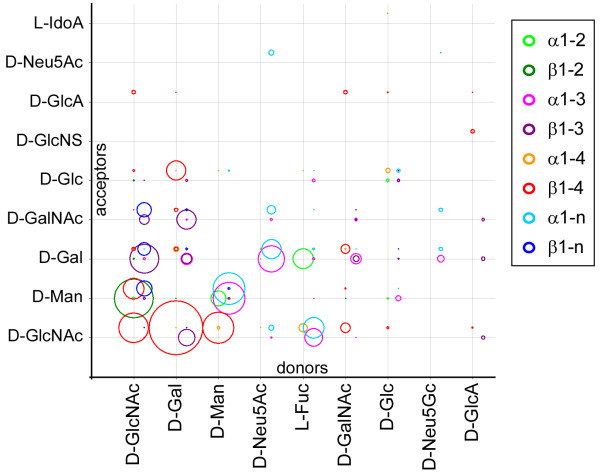
**Glycosidic linkages in mammalian carbohydrates**. Frequency distribution of specific disaccharide linkages in mammalian carbohydrates. Plotted circle areas represent the relative frequencies for disaccharides formed from the 9 most common donors (children) and 9 most common acceptors (parents) in a defined glycosidic linkage (color code in legend). The areas of the circles are proportional to the relative abundances of disaccharide pairs, normalized to the total number of specific disaccharide pairs. The linkage codes α1-n and β1-n correspond to a linkage to any exocyclic carbon at the acceptor, e.g. C6 in hexopyranoses. For donor residues in keto form the linkage is at the anomeric carbon C2 instead of C1. For better visualization some of the circles for a given linkage are offset somewhat from the matrix coordinate corresponding to a given linkage type.

The analysis summarized in Fig. 11 demonstrates that the most prevalent linkages in mammals are D-Gal, D-Man and D-GlcNAc as β1–4 donors to D-GlcNAc; D-GlcNAc as β1–2 donor to D-Man; D-GlcNAc as β1–3 donor to D-Gal; and D-Man disaccharides with α1–3 or α1–6 linkages. These monosaccharides are sufficient to build up the common *N*- and *O-*glycan structures which dominate the mammalian database.

A complete list of mammalian disaccharide fragments found in GLYCOSCIENCES.de is available [see Additional file 5], where the data are encoded to illustrate the differences between our database findings and the data from Ohtsubo & Marth [2]. Three disaccharides reported in [2], namely D-Glc(α1–2) D-Gal, D-GlcA(β1–4) D-Gal and D-GlcNAc(α1–6) D-GlcNAc, are absent from our databases. On the other hand, many existing mammalian disaccharides have not been mentioned by Ohtsubo & Marth, among them reasonably abundant ones such as D-GlcNAc(β1–3) D-GalNAc, D-GlcNAc(β1–4) D-Man, D-GlcNAc(β1–6) D-Man, and D-GlcNS(α1–4) L-IdoA. The last disaccharide listed is present in more than 500 records of GLYCOSCIENCES.DE for human carbohydrates and reported in the literature in association with Sandhoff's Disease [25].

The disaccharide ensemble or total count of unique linkages is considerably larger for most of the bacterial classes compared to mammals, as shown in Table 4. For this analysis we have taken a total of 24613 bacterial and 23883 mammalian disaccharide fragments into account.

**Table 4:** Diversity of disaccharide linkages found in various taxonomic groups.

**Group**	**Disaccharide Linkages**
*Actinobacteria*	364
*Bacilli*	526
α-*Proteobacteria*	319
β-*Proteobacteria*	356
*Enterobacteriales *(γ-*Proteobacteria*)	1570
other γ-*Proteobacteria*	1148
δ/ε-*Proteobacteria*	218
*Mammalia*	488

Total count of all unique disaccharide linkages per taxonomic group. Unknown anomeric and absolute configurations and fuzzily defined linkages are counted as distinct entities.

The abundance data for the more frequently occurring disaccharide linkage patterns found in mammals and/or bacteria are presented in Table 5 (detected in the combined database using an abundance threshold = 0.1% for either mammals or bacteria). More comprehensive data (with lower threshold) are presented in the additional material [see Additional file 6]. In Table 5 there are 26 disaccharide linkages listed which are found only in bacteria. The four most frequent of these, with abundances of 0.40–0.68% (underlined in Table 5) are D-Glc(α1–4) D-Gal, D-Gal(α1–2) D-Gal, D-Glc(b1–4) D-Gal, and D-Gal(α1–2) D-Man. In Table 5 there are five disaccharide types which occur in mammals only, and the most abundant of these (underlined in Table 5) involve (a) α2–6 linkages from a neuraminic acid (Neu5Ac or Neu5Gc) to D-GalNAc or (b) α1–4 linkages from a uronic acid (GlcA, IdoA, or ΔGlcA) to D-GlcN-sulfate.

**Table 5:** Identity and abundances of mammalian and bacterial disaccharides.

	**Acceptors (parents)**									
	
**Donors (children)**	**Fuc**	**Gal**	**GalNAc**	**Glc**	**GlcNAc**	**GlcA, IdoA or ΔGlcA**	**Man**	**Neu5Ac or Neu5Gc**	**Xyl**	**GlcNS**
**Fuc**	a1–3 (0.01) [0.15]	a1–2 (2.68) [0.18]			a1–3 (2.00) [0.32]					
					a1–4 (0.54) [0.09]					
					a1–6 (2.84) [0.01]					

**Gal**		a1–3 (0.92) [0.60]	a1–3 (0.02) [0.12]	a1–3 (0.00) [0.32]	a1–3 (0.00) [0.26]		a1–2 [0.40]		b1–4 (0.11) [0.00]	
		a1–4 (0.22) [0.54]	b1–3 (2.41) [0.54]	a1–6 (0.00) [0.49]	b1–3 (1.86) [0.52]		a1–3 [0.10]			
		a1–6 (0.02) [0.25]	b1–4 (0.11) [0.11]	b1–3 (0.04) [0.18]	b1–4 (18.06) [1.68]		a1–6 [0.12]			
		b1–3 (0.70) [0.80]		b1–4 (2.51) [2.32]						
		b1–4 (0.11) [0.34]		b1–6 (0.00) [0.18]						
		b1–6 (0.05) [0.42]		a1–2 [0.25]						
		a1–2 [0.52]								

**GalNAc**		a1–3 (0.80) [0.05]	a1–3 (0.07) [0.32]		b1–4 (0.68) [0.02]	b1–4 (0.12) [0.08]				
		b1–3 (0.30) [0.44]	b1–3 (0.03) [0.10]							
		b1–4 (0.61) [0.36]	a1–4 [0.14]							
			b1–4 [0.20]							
**Glc**		b1–3 (0.02) [0.40]	a1–6 [0.12]	a1–2 (0.06) [0.95]	b1–3 [0.11]	a1–4 (0.03) [0.27]	a1–3 (0.19) [0.19]			
		b1–6 (0.01) [0.34]	b1–3 [0.18]	a1–3 (0.08) [0.83]	b1–6 [0.15]	b1–4 (0.01) [0.12]	b1–4 (0.00) [0.14]			
		a1–2 (*) [0.31]		a1–4 (0.15) [0.39]		b1–3 [0.10]				
		a1–3 [0.13]		a1–6 (0.10) [0.57]						
		a1–4 [0.68]		b1–3 (0.05) [0.62]						
		a1–6 [0.13]		b1–4 (0.00) [1.69]						
		b1–4 [0.40]		b1–6 (0.01) [0.90]						
				a1–1 [0.16]						
				b1–2 [0.33]						

**GlcNAc**		b1–3 (5.38) [1.64]	b1–3 (0.68) [0.04]		b1–3 (0.01) [0.34]	a1–4 (0.10) [0.05]	b1–2 (9.46) [0.11]			
		b1–4 (0.13) [0.02]	b1–6 (1.45) [0.00]		b1–4 (5.61) [0.69]	b1–4 (0.13) [0.13]	b1–4 (2.83) [0.05]			
		b1–6 (1.17) [0.04]			b1–6 (0.01) [0.22]		b1–6 (1.64) [0.02]			
					b1–2 [0.15]					

**GlcA, IdoA or ΔGlcA**		b1–3 (0.13) [0.23]	a1–3 (0.14) [0.01]	b1–4 [0.26]	a1–4 (0.16) [0.01]	b1–4 (0.01) [0.19]	a1–3 [0.22]			a1–4 (0.44)
		b1–4 (*) [0.14]			b1–3 (0.13) [0.14]	b1–3 [0.13]	b1–2 [0.19]			b1–4 (0.10)

**Man**		a1–3 [0.22]		a1–3 (0.00) [0.27]	b1–4 (6.22) [0.02]		a1–2 (1.60) [0.92]			
							a1–3 (6.39) [0.41]			
							a1–4 (0.01) [0.16]			
							a1–6 (6.35) [0.53]			
							b1–4 [0.17]			

**Neu5Ac or Neu5Gc**		a2–3 (4.85) [1.01]	a2–6 (0.49)		a2–6 (0.22)			a2–8 (0.29) [0.12]		
		a2–6 (2.64) [0.02]								

**Xyl**									b1–4 (0.00) [0.10]	

**GlcNS**						a1–4 (0.34)				

Linkage types and relative abundances are shown as percentages (rounded to two decimals) of the total disaccharide fragment count for mammals (values in parentheses) or bacteria [values in brackets]. Linkages are listed only for disaccharide fragments present at > 0.1% in either taxonomy group. Values of 0.00 indicate the presence of a linkage at < 0.005%; missing values indicate the absence of that linkage type in the corresponding taxonomy group. An entry (*) denotes that the linkage was reported for mammals in [2] but was not found in the databases. The percentages are cumulative for all ring types (including alditols), all absolute configurations and for all anomeric configurations of the acceptor. For linkages that occur only in mammals or only in bacteria, those with abundences of 0.40% or more are underlined.

**Figure 12:**
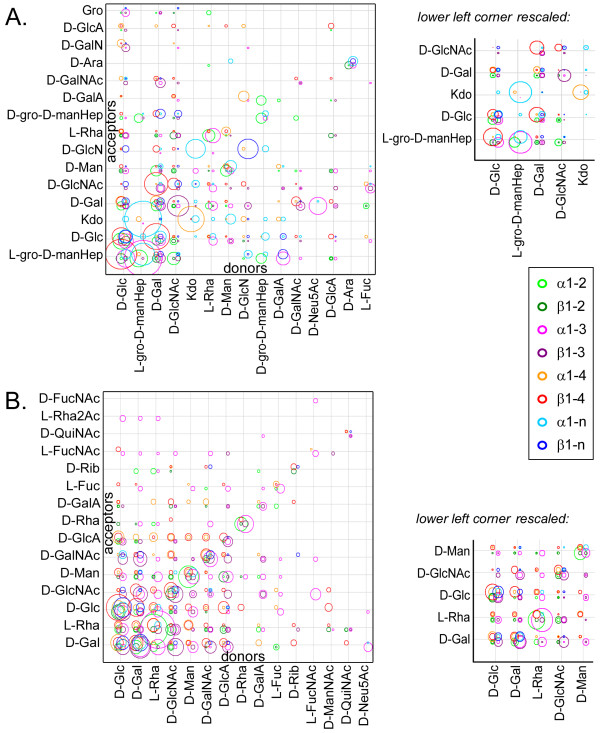
**Glycosidic linkages in bacterial carbohydrates**. Frequency distribution of specific disaccharide linkages in bacterial carbohydrates. Plotted circle areas represent the relative frequencies for disaccharides formed from the 15 most common donors (children) and 15 most common acceptors (parents) in a defined glycosidic linkage (color code in legend) for bacterial oligomers (**A**) or polymers (**B**). The areas of the circles are proportional to the relative abundances of specific disaccharide pairs, normalized to the total number of disaccharide pairs. The linkage codes and plotting offsets are used as in Fig. 12. The lower left corner of each diagram is plotted at the right with rescaling for better visualization.

The abundances of the most common bacterial disaccharide fragments are shown schematically in Fig. 12, where separate diagrams are presented for oligomers (A) and polymers (B) using the same color-coded linkage scheme as in Fig. 11. The residue names are sorted according to their abundance either as donors (children) or acceptors (parents). The highest abundances in bacterial oligomers (Fig. 12A) are exhibited by the constituents of the bacterial lipopolysaccharide core region: L-gro-D-manHep→Kdo and L-gro-D-manHep→L-gro-D-manHep. High abundances for L-Rha→L-Rha, D-Man→D-Man, D-Gal→D-Gal and D-Glc→D-Glc in Fig. 12B arise from the homopolymeric regions prevalent in bacterial polymers.

A potential application of the information and methods outlined here is the design and validation of carbohydrate vaccines against bacterial pathogens. Carbohydrate-based vaccines against *Haemophilus influenzae *Type b, *Neisseria meningitidis *and *Streptococcus pneumoniae *have already been licensed, and many similar products are in various stages of development. For example, the disaccharides D-Glc(α1–2)D-Gal and D-Glc(β1–4)D-Gal are not present in mammalian organisms accordingly to our analysis and are both constituents of the capsular polysaccharides of *Salmonella pneumonia*, which were shown to be target candidates for vaccine development [26].

## Conclusion

In this study we combined the BCSDB, the largest available bacterial carbohydrate database, with the GLYCOSCIENCES.de database to obtain a set of 13775 nonredundant glycan/taxon pairs (carbohydrate sequences with a defined taxonomy), of which 6098 were assigned to *Bacteria *and 4739 to *Mammalia*. The representative statistical analyses presented here reveal the basic principles of carbohydrate architecture in bacteria vs. mammals. The major monosaccharides which characterize different branches of the tree of life were extracted from the database and are in accordance with the published literature. Several monosaccharides unique to certain subclasses of bacteria were identified and could prove useful as molecular markers for these classes. Similarly, a variety of structural modifications of monosaccharides have been detected, and many of these are characteristic in that they may be either highly abundant or totally absent in individual taxonomic classes.

A linkage analysis was performed for all disaccharide fragments of bacterial and mammalian glycans and revealed that there are a number of abundant linkages as well as nonexistent linkages which may be useful for characterizing the various taxonomic groups. Through a comparison of the disaccharide linkage ensembles or spaces for bacteria and mammals, one obtains an overview of those glycosyltransferase activities which are common to both classes and those which appear to be unique for mammals or bacteria or even for specific bacteria subclasses. Thus, differential cross-species expression analysis is possible and may ultimately provide a deeper understanding of immunogenic patterns present in pathogenic bacteria.

## Methods

The analyzed sequences were obtained from the meta-database GlycomeDB [27], which contains all sequences from the Bacterial Carbohydrate Structure DataBase (BCSDB) [12] and the GLYCOSCIENCES.de portal [13] in a harmonized format. With the help of the NCBI taxonomy database [15], subsets of these databases were taken and further analyzed using routines implemented in JAVA. The results of the analytic routines were stored in a PostgreSQL 8.2 database. Additional analytical procedures were implemented in PHP, which finally generated Microsoft Excel tables used for further analysis and graphical visualization.

### Definition of terms

According to IUPAC nomenclature a *monosaccharide *is a poly(hydroxy) aldehyde or ketone with three or more carbon atoms (triose, tetrose, etc.); the term denotes a single structural component without glycosidic linkages and includes a variety of derivatives such as amino, deoxy or carboxy forms. *Oligosaccharides *are compounds in which monosaccharides and their derivatives are coupled in a precisely defined manner via glycosidic linkages. The term *polysaccharide *generally refers to oligosaccharides with a large or undefined number of monosaccharide residues. The term *carbohydrate *includes all mono-, oligo- or polysaccharides and molecules derived from monosaccharides modified by reduction, oxidation, or substitution. The term *glycan *is frequently used to refer to any saccharide component of a glycoconjugate, such as a glycoprotein or glycolipid, even when the chain length is short. A *glycoconjugate *is formed by a covalent linkage between a glycan and a nonglycan entity. *Polysaccharide *may be used to refer to polymers with glycosidic and/or phosphodiester linkages (such as teichoic acids). The carbohydrate databases used in this study may contain any of the compounds described above but do not contain DNAs or RNAs.

In this study we used the following definitions:

1. The term *sequence *will be used here to refer to a specific carbohydrate molecule or a glycan obtained from a larger molecule (glycoconjugate). Sequences may be linear or branched. Each database record may refer to either an individual carbohydrate sequence or to a particular glycoconjugate containing a given glycan. Thus, each unique glycan may have multiple database records, one for each different glycoconjugate.

2. A *residue *is a specific building block, e.g. a monosaccharide, within a carbohydrate sequence, analogous to the amino acid residues in proteins.

3. The term *unit *will be used to specify the smallest sequence fragment which describes a given carbohydrate molecule or glycan. For a nonrepeating oligomer sequence the unit will be the entire sequence; for polymers built up from repeating subsequences, the unit will be one such subsequence.

4. A *branching point *is a particular residue to which *two or more *carbohydrate residues are attached via nonreducing hydroxy functions or other functional groups.

5. A *monosaccharide *is a unique carbohydrate residue according to the IUPAC definition given above and is specified by the number of carbons, the ring type, the anomeric (α,β) and absolute (D, L) configurations, and all primary and secondary modifications. For example, α-D-Glc*p*N, β-D-Glc*p*N and α-D-Glc*p*NAc are three different monosaccharides derived from glucose.

6. *Primary modifications *of monosaccharides are those which alter the stereochemical designation or electronic hybridisation state of at least one carbon atom (e.g. deoxy, carboxy, keto, double-bond modifications). *Secondary modifications *are all modifications which are not primary (substituents such as amino, *O*-methyl, *O*-acetyl, sulfate, phosphate, etc.).

7. We define the *basetype *of a monosaccharide to include only those characteristics which specify the order and stereochemical designations of its carbon atom skeleton, i.e., the anomeric and absolute configurations, ring type, and primary modifications. *Basetype *is *not *altered by secondary modifications. Thus, α-D-Glc*p*, α-D-Glc*p*N and α-D-Glc*p*6S all have the same basetype (α-D-Glc*p*) while β-D-Glc*p *and α-D-Glc*p*A are different basetypes.

8. With respect to common historical usage, the term *basic entity *will be used to specify the following characteristics of a monosaccharide: the stereochemical configuration (D, L), all primary modifications, and only those secondary modifications involving amine groups, including substituted amines, at any position other than the anomeric carbon. The basic entity definition does *not *include anomeric configuration, ring type, or any secondary modifications. Thus, α-D-Gal*f*N and β-D-Gal*p*NAc have the same basic entity (D-GalN) while β-D-Gal*p*, β-D-Gal*p*A and β-D-Fuc*p *are all different basic entities.

## Authors' contributions

SH carried out the data generation, programmed the analytical procedures and drafted the manuscript together with PVT, who made the statistical analyses and the figures. RR participated in the data generation and made significant contributions to the programming framework. C–WvdL, WEH and YAK participated in the design of the study and helped to draft the manuscript. All authors read and approved the final manuscript.

## Supplementary Material

Additional file 1 Supplementary Table 1: Abundance of carbohydrate sequences for various taxonomical classes, orders and families. Click here for file

Additional file 2 Supplementary Table 2: Abundance of basetypes for bacterial and mammalian carbohydrates. Click here for file

Additional file 3 Supplementary Table 3: Abundances of monosaccharide residues found in bacterial and mammalian carbohydrates. Click here for file

Additional file 4 Supplementary Table 4: Abundances of all monosaccharide pairs found in bacterial and mammalian carbohydrates. Click here for file

Additional file 5 Supplementary Table 5: Comprehensive list of mammalian disaccharide fragments and their relative abundances. Click here for file

Additional file 6 Supplementary Table 6: Comparison of mammalian and bacterial disaccharide fragments and their relative abundances. Click here for file
