# Supplementary material for: Statistical analysis of the Bacterial Carbohydrate Structure Data Base (BCSDB): Characteristics and diversity of bacterial carbohydrates in comparison with mammalian glycans
Source: BMC Struct Biol. 2008 Aug 11;8:35. doi: 10.1186/1472-6807-8-35 (PMC2543016; doi:10.1186/1472-6807-8-35)
Supplement: Additional file 5 — Supplementary Table 5: Comprehensive list of mammalian disaccharide fragments and their relative abundances. [file 1472-6807-8-35-S5.rtf]

Supplementary Table 5. Linkage types for mammalian disaccharide fragments with more than 3 occurrences in the GLYCOSCIENCES.de database. Relative abundances are in parentheses (in percent, rounded to two decimals, relative to total disaccharide fragment count). Values of 0.00 denote a frequency of <0.005%. The percentages are cumulative for all ring types (including alditols), all absolute configurations and for all anomeric configurations of the acceptor. Linkage codes in ( ) were reported by Ohtsubo & Marth in [2] but were not above the threshold in our database. Entries in italic font were found in the database and in [2]; entries in normal font were not found in [2] and the most abundant of those (>0.1%) are listed in bold face.
Donors (children )	Acceptors (parents)	
	Fuc	Gal	GalNAc	Glc	GlcNAc	GlcA, IdoA or threo-HexA-ä4,5	Man	Neu5Ac	Xyl	Neu5Gc	GlcNS	
Fuc	a1-2 (0.00)
a1-3 (0.01)	a1-2 (2.68)
a1-3 (0.04)
a1-6 (0.04)
b1-3 (0.00)	a1-6 (0.01)	a1-2 (0.00)
a1-3 (0.09)
a1-4 (0.01)
a1-6 (0.01)	a1-3 (2.00)
a1-4 (0.54)
a1-6 (2.84)
b1-3 (0.00)		a1-2 (0.00)
a1-3 (0.01)					
Gal	b1-4 (0.00)	a1-3 (0.92)
a1-4 (0.22)
a1-6 (0.02)
b1-3 (0.70)
b1-4 (0.11)
b1-6 (0.05)	a1-3 (0.02)
b1-3 (2.41)
b1-4 (0.11)
b1-6 (0.04)	a1-3 (0.00)
a1-4 (0.01)
a1-6 (0.00)
b1-3 (0.04)
b1-4 (2.51)
b1-6 (0.00)	a1-3 (0.00)
a1-4 (0.01)
b1-3 (1.86)
b1-4 (18.06)
b1-6 (0.01)	b1-4 (0.01)	b1-2 (0.01)		b1-3 (0.03)
b1-4 (0.11)			
GalNAc		a1-3 (0.80)
a1-4 (0.02)
a1-6 (0.01)
b1-3 (0.30)
b1-4 (0.61)
b1-6 (0.00)	a1-3 (0.07)
a1-6 (0.01)
b1-3 (0.03)
b1-6 (0.01)		a1-3 (0.01)
b1-4 (0.68)	a1-4 (0.00)
b1-4 (0.12)	b1-2 (0.01)
b1-4 (0.02)
b1-6 (0.00)					
Glc	b1-3 (0.00)	b1-2 (0.00)
b1-3 (0.02)
b1-6 (0.01)
(a1-2)	a1-4 (0.00)	a1-2 (0.06)
a1-3 (0.08)
a1-4 (0.15)
a1-6 (0.10)
b1-3 (0.05)
b1-4 (0.00)
b1-6 (0.01)	a1-4 (0.01)
b1-4 (0.06)	a1-4 (0.03)
b1-4 (0.01)	a1-2 (0.02)
a1-3 (0.19)
a1-6 (0.01)
b1-2 (0.00)
b1-4 (0.00)
b1-6 (0.00)					
GlcNAc	b1-3 (0.01)	a1-3 (0.04)
a1-4 (0.08)
a1-6 (0.01)
b1-2 (0.03)
b1-3 (5.38)
b1-4 (0.13)
b1-6 (1.17)	b1-3 (0.68)
b1-4 (0.01)
b1-6 (1.45)	b1-2 (0.03)
b1-3 (0.01)
b1-4 (0.03)	b1-3 (0.01)
b1-4 (5.61)
b1-6 (0.01)
(a1-6)	a1-4 (0.10)
b1-4 (0.13)	a1-2 (0.07)
b1-2 (9.46)
b1-3 (0.03)
b1-4 (2.83)
b1-6 (1.64)					


Supplementary Table 5. (continued)

Donors (children )	Acceptors (parents)	
	Fuc	Gal	GalNAc	Glc	GlcNAc	GlcA, IdoA or threo-HexA-ä4,5	Man	Neu5Ac	Xyl	Neu5Gc	GlcNS	
GlcA, IdoA or threo-HexA-ä4,5		a1-3 (0.01)
b1-3 (0.13)
(b1-4)	a1-3 (0.14)
a1-4 (0.00)
b1-3 (0.07)	a1-4 (0.00)	a1-4 (0.16)
b1-3 (0.13)
b1-4 (0.03)	a1-4 (0.00)
b1-4 (0.01)			b1-4 (0.01)		a1-4 (0.44)
b1-4 (0.10)	
Man				a1-3 (0.00)
a1-4 (0.01)
a1-6 (0.02)	a1-4 (0.08)
b1-4 (6.22)		a1-1 (0.00)
a1-2 (1.60)
a1-3 (6.39)
a1-4 (0.01)
a1-6 (6.35)
b1-3 (0.04)
b1-6 (0.04)					
Neu5Ac		a2-2 (0.01)
a2-3 (4.54)
a2-4 (0.03)
a2-6 (2.53)
b2-3 (0.00)
b2-6 (0.00)	a2-3 (0.03)
a2-6 (0.48)		a2-3 (0.02)
a2-4 (0.01)
a2-6 (0.22)		a2-6 (0.00)	a2-7 (0.00)
a2-8 (0.22)
a2-9 (0.00)		a2-8 (0.03)		
Xyl				a1-3 (0.01)			b1-2 (0.03)		a1-3 (0.00)
b1-4 (0.00)			
Neu-5Gc		a2-3 (0.31)
a2-6 (0.11)	a2-6 (0.10)		a2-6 (0.00)			a2-8 (0.01)		a2-8 (0.03)		
Glc-NS						a1-4 (0.34)
b1-4 (0.00)					a1-4 (0.00)	
