# Supplementary material for: Statistical analysis of the Bacterial Carbohydrate Structure Data Base (BCSDB): Characteristics and diversity of bacterial carbohydrates in comparison with mammalian glycans
Source: BMC Struct Biol. 2008 Aug 11;8:35. doi: 10.1186/1472-6807-8-35 (PMC2543016; doi:10.1186/1472-6807-8-35)
Supplement: Additional file 6 — Supplementary Table 6: Comparison of mammalian and bacterial disaccharide fragments and their relative abundances. [file 1472-6807-8-35-S6.rtf]

Supplementary Table 6. Linkage types for mammalian and bacterial disaccharides with more than 3 occurrences in the combined databases. Relative abundances are in parentheses (mammals) or in square brackets (bacteria) and are expressed as percentages relative to the total count of disaccharide fragments in the corresponding taxonomic group. The presentation is analogous to the main Table 5 (see Legend there for details) but with a lower frequency threshold. 
Donors (children)	Acceptors (parents)	
	Fuc	Gal	GalNAc	Glc	GlcNAc	GlcA, IdoA
or ÄGlcA	Man	Neu5Ac or Neu5Gc	Xyl	GlcNS	
Fuc	a1-2 (0.00) [0.03]
a1-3 (0.01) [0.15]
a1-4 [0.06]
a1-6 [0.00]
b1-4 [0.01]	a1-2 (2.68) [0.18]
a1-3 (0.04) [0.01]
a1-6 (0.04) [0.01]
b1-3 (0.00)
a1-4 [0.00]
b1-2 [0.02]	a1-6 (0.01)
a1-3 [0.04]
a1-4 [0.00]	a1-2 (0.00)
a1-3 (0.09) [0.09]
a1-4 (0.01) [0.05]
a1-6 (0.01) [0.00]
b1-2 [0.00]
b1-4 [0.00]	a1-3 (2.00) [0.32]
a1-4 (0.54) [0.09]
a1-6 (2.84) [0.01]
b1-3 (0.00) [0.07]	a1-2 [0.03]
a1-3 [0.00]
a1-4 [0.00]	a1-2 (0.00) [0.04]
a1-3 (0.01)
a1-4 [0.00]
a1-6 [0.01]				
Gal	b1-4 (0.00) [0.02]
a1-2 [0.01]
a1-3 [0.07]
b1-3 [0.00]	a1-3 (0.92) [0.60]
a1-4 (0.22) [0.54]
a1-6 (0.02) [0.25]
b1-3 (0.7) [0.80]
b1-4 (0.11) [0.34]
b1-6 (0.05) [0.42]
a1-1 [0.01]
a1-2 [0.52]
b1-2 [0.09]
b1-5 [0.07]	a1-3 (0.02) [0.12]
b1-3 (2.41) [0.54]
b1-4 (0.11) [0.11]
b1-6 (0.04) [0.07]
a1-4 [0.04]	a1-3 (0.00) [0.32]
a1-4 (0.01) [0.08]
a1-6 (0.00) [0.49]
b1-3 (0.04) [0.18]
b1-4 (2.51) [2.32]
b1-6 (0.00) [0.18]
a1-2 [0.25]
b1-2 [0.01]	a1-3 (0.00) [0.26]
a1-4 (0.01) [0.01]
b1-3 (1.86) [0.52]
b1-4 (18.06) [1.68]
b1-6 (0.01) [0.02]
a1-6 [0.06]	b1-4 (0.01) [0.00]
a1-2 [0.02]
a1-4 [0.07]
b1-2 [0.01]
b1-3 [0.02]	b1-2 (0.01) [0.02]
a1-2 [0.4]
a1-3 [0.1]
a1-4 [0.02]
a1-6 [0.12]
b1-1 [0.00]
b1-3 [0.02]
b1-4 [0.03]
b1-6 [0.04]	a1-4 [0.02]	b1-3 (0.03)
b1-4 (0.11) [0.00]
a1-4 [0.00]		
GalNAc	a1-2 [0.01]
a1-3 [0.00]	a1-3 (0.80) [0.05]
a1-4 (0.02) [0.03]
a1-6 (0.01) [0.00]
b1-3 (0.30) [0.44]
b1-4 (0.61) [0.36]
b1-6 (0.00) [0.03]
a1-2 [0.00]
b1-2 [0.01]	a1-3 (0.07) [0.32]
a1-6 (0.01) [0.02]
b1-3 (0.03) [0.1]
b1-6 (0.01) [0.08]
 a1-2 [0.02]
a1-4 [0.14]
a1-7 [0.00]
b1-2 [0.07]
b1-4 [0.20]
b1-7 [0.01]	a1-4 [0.01]
a1-6 [0.02]
b1-2 [0.01]
b1-3 [0.02]
b1-4 [0.07]
b1-6 [0.00]	a1-3 (0.01) [0.08]
b1-4 (0.68) [0.02]
a1-6 [0.02]
b1-3 [0.02]
b1-6 [0.01]	a1-4 (0.00) [0.03]
b1-4 (0.12) [0.08]
b1-3 [0.00]	b1-2 (0.01) [0.00]
b1-4 (0.02) [0.01]
b1-6 (0.00)
a1-2 [0.02]
a1-3 [0.05]
a1-4 [0.01]	a1-4 [0.00]
b1-4 [0.01]
b1-7 [0.00]			
Glc	b1-3 (0.00) [0.01]
a1-3 [0.00]
b1-4 [0.07]	b1-2 (0.00) [0.06]
b1-3 (0.02) [0.40]
b1-6 (0.01) [0.34]
a1-2 (*) [0.31]
a1-3 [0.13]
a1-4 [0.68]
a1-6 [0.13]
b1-4 [0.40]
b1-5 [0.03]	a1-4 (0.00) [0.06]
a1-3 [0.00]
a1-6 [0.12]
b1-3 [0.18]
b1-5 [0.01]
b1-6 [0.03]	a1-2 (0.06) [0.95]
a1-3 (0.08) [0.83]
a1-4 (0.15) [0.39]
a1-6 (0.10) [0.57]
b1-3 (0.05) [0.62]
b1-4 (0.00) [1.69]
b1-6 (0.01) [0.90]
a1-1 [0.16]
b1-1 [0.01]
b1-2 [0.33]	a1-4 (0.01) [0.02]
b1-4 (0.06) [0.02]
a1-3 [0.01]
a1-6 [0.05]
b1-3 [0.11]
b1-6 [0.15]	a1-4 (0.03) [0.27]
b1-4 (0.01) [0.12]
a1-2 [0.05]
a1-3 [0.02]
b1-2 [0.01]
b1-3 [0.10]	a1-2 (0.02) [0.02]
a1-3 (0.19) [0.19]
a1-6 (0.01)
b1-2 (0.00) [0.08]
b1-4 (0.00) [0.14]
b1-6 (0.00) [0.01]
a1-4 [0.01]
b1-3 [0.02]	a1-4 [0.00]
b1-7 [0.01]	b1-4 [0.02]		


Supplementary Table 6. (continued)

Donors (children)	Acceptors (parents)	
	Fuc	Gal	GalNAc	Glc	GlcNAc	GlcA, IdoA
or ÄGlcA	Man	Neu5Ac or Neu5Gc	Xyl	GlcNS	
GlcNAc	b1-3 (0.01) [0.00]
a1-3 [0.03]
b1-2 [0.01]
b1-4 [0.03]	a1-3 (0.04) [0.07]
a1-4 (0.08) [0.02]
a1-6 (0.01)
b1-2 (0.03) [0.09]
b1-3 (5.38) [1.64]
b1-4 (0.13) [0.02]
b1-6 (1.17) [0.04]
a1-2 [0.08]
b1-5 [0.00]	b1-3 (0.68) [0.04]
b1-4 (0.01) [0.07]
b1-6 (1.45) [0.00]
a1-3 [0.01]
a1-4 [0.09]
a1-6 [0.01]	b1-2 (0.03) [0.07]
b1-3 (0.01) [0.03]
b1-4 (0.03) [0.05]
a1-2 [0.20]
a1-3 [0.01]
a1-4 [0.02]
a1-6 [0.00]
b1-6 [0.04]	b1-3 (0.01) [0.34]
b1-4 (5.61) [0.69]
b1-6 (0.01) [0.22]
a1-6 [0.05]
a1-2 [0.05]
a1-3 [0.07]
a1-4 [0.08]
a1-8 [0.00]
b1-2 [0.15]
b1-5 [0.00]
b1-7 [0.00]	a1-4 (0.10) [0.05]
b1-4 (0.13) [0.13]
a1-3 [0.01]
b1-2 [0.01]
b1-3 [0.03]	a1-2 (0.07) [0.06]
b1-2 (9.46) [0.11]
b1-3 (0.03)
b1-4 (2.83) [0.05]
b1-6 (1.64) [0.02]
a1-6 [0.02]	b1-7 [0.02]			
GlcA, IdoA
or ÄGlcA	a1-3 [0.05]
a1-4 [0.01]
b1-4 [0.04]	a1-3 (0.01) [0.07]
b1-3 (0.13) [0.23]
b1-4 (*) [0.14]
a1-2 [0.01]
a1-4 [0.02]
b1-6 [0.02]	a1-3 (0.14) [0.01]
a1-4 (0.00) [0.01]
b1-3 (0.07) [0.09]
b1-4 [0.05]
b1-6 [0.01]	a1-4 (0.00) [0.01]
a1-3 [0.03]
a1-6 [0.01]
b1-3 [0.01]
b1-4 [0.26]
b1-6 [0.02]	a1-4 (0.16) [0.01]
b1-3 (0.13) [0.14]
b1-4 (0.03) [0.02]
a1-3 [0.00]
a1-4 [0.01]
a1-6 [0.01]	a1-4 (0.00) [0.01]
b1-4 (0.01) [0.19]
a1-2 [0.02]
a1-3 [0.05]
b1-3 [0.13]
b1-6 [0.01]	a1-2 [0.02]
a1-3 [0.22]
b1-2 [0.19]
b1-3 [0.02]		b1-4 (0.01)	a1-4 (0.44)
b1-4 (0.10)	
Man	a1-2 [0.00]
a1-3 [0.04]
a1-4 [0.01]	a1-2 [0.01]
a1-3 [0.22]
a1-4 [0.07]
b1-3 [0.03]
b1-4 [0.05]	a1-3 [0.02]
b1-3 [0.01]
b1-4 [0.01]	a1-3 (0.00) [0.27]
a1-4 (0.01) [0.01]
a1-6 (0.02)
b1-3 [0.07]
b1-4 [0.07]
b1-6 [0.00]	a1-4 (0.08)
b1-4 (6.22) [0.02]
a1-3 [0.02]
b1-3 [0.09]	a1-4 [0.03]
b1-4 [0.09]	a1-1 (0.00)
a1-2 (1.60) [0.92]
a1-3 (6.39) [0.41]
a1-4 (0.01) [0.16]
a1-6 (6.35) [0.53]
b1-3 (0.04) [0.07]
b1-6 (0.04)
b1-2 [0.07]
b1-4 [0.17]		a1-3 [0.01]		
Neu5Ac or Neu5Gc		a2-2 (0.01)
a2-3 (4.85) [1.01]
a2-4 (0.03)
a2-6 (2.64) [0.02]
b2-3 (0.00)
b2-6 (0.00)	a2-3 (0.03)
a2-6 (0.49)	a1-6 [0.00]
a2-3 [0.02]
a2-6 [0.00]
b2-3 [0.00]	a2-3 (0.02)
a2-4 (0.01)
a2-6 (0.22)		a2-6 (0.00)	a2-7 (0.00)
a2-8 (0.29) [0.12]
a2-9 (0.00) [0.03]
a2-3 [0.02]
a2-6 [0.01]			
Xyl	b1-4 [0.01]			a1-3 (0.01) 
b1-3 [0.01]
b1-4 [0.01]	b1-4 [0.00]	b1-3 [0.01]	b1-2 (0.03)
a1-4 [0.00]		a1-3 (0.00) [0.03]
b1-4 (0.00) [0.10]		
GlcNS						a1-4 (0.34)
b1-4 (0.00)				a1-4 (0.00)	
